# Molecular and cellular characteristics of cerebrovascular cell types and their contribution to neurodegenerative diseases

**DOI:** 10.1186/s13024-025-00799-z

**Published:** 2025-01-29

**Authors:** Francisco J. Garcia, Myriam Heiman

**Affiliations:** 1 The Picower Institute for Learning and Memory, Cambridge, MA USA; 2 Department of Brain and Cognitive Sciences, MIT, Cambridge, MA USA

**Keywords:** Cerebrovasculature, Brain vasculature, Neurodegenerative diseases, Enhanced vulnerability, Blood-brain barrier, Neurovascular unit, Transcriptional dysregulation, snRNA-seq, Cell type-specificity

## Abstract

Many diseases and disorders of the nervous system suffer from a lack of adequate therapeutics to halt or slow disease progression, and to this day, no cure exists for any of the fatal neurodegenerative diseases. In part this is due to the incredible diversity of cell types that comprise the brain, knowledge gaps in understanding basic mechanisms of disease, as well as a lack of reliable strategies for delivering new therapeutic modalities to affected areas. With the advent of single cell genomics, it is now possible to interrogate the molecular characteristics of diverse cell populations and their alterations in diseased states. More recently, much attention has been devoted to cell populations that have historically been difficult to profile with bulk single cell technologies. In particular, cell types that comprise the cerebrovasculature have become increasingly better characterized in normal and neurodegenerative disease contexts. In this review, we describe the current understanding of cerebrovasculature structure, function, and cell type diversity and its role in the mechanisms underlying various neurodegenerative diseases. We focus on human and mouse cerebrovasculature studies and discuss both origins and consequences of cerebrovascular dysfunction, emphasizing known cell type-specific vulnerabilities in neuronal and cerebrovascular cell populations. Lastly, we highlight how novel insights into cerebrovascular biology have impacted the development of modern therapeutic approaches and discuss outstanding questions in the field.

## Introduction

Aging-associated neurodegenerative diseases exhibit distinct patterns of enhanced vulnerability, whereby specific populations of neurons are earliest and most affected in each disease [1]. Disease symptoms and progression, in turn, reflect the dysfunction of these most vulnerable cell types and associated circuits. In many cases, it is not entirely clear what underlies the basis of this enhanced vulnerability in neurodegenerative disease. Even for monogenic diseases, such as Huntington’s disease (HD) and the spinocerebellar ataxias (SCAs), where the causal genes have been known for decades [2, 3], the precise mechanisms underlying neurodegeneration and disease progression are not completely understood.

Cell type composition in the brain is diverse and varies greatly by region. Many studies have applied modern single cell and single nucleus RNA-sequencing (scRNA-seq and snRNA-seq) approaches to tissues from mouse and human samples in order to atlas this diversity of cell populations of the mammalian brain and gain insights into the molecular basis underlying cell structure and function [4–7]. Furthermore, single cell studies of specific neurodegenerative diseases have focused on profiling cells in the affected brain regions to understand cell type-specific dysregulation with the potential of uncovering disease-relevant gene expression patterns that could lead into the development of novel therapeutic strategies [8–12]. These studies have highlighted the interplay between affected neurons and other cell types of the brain, especially with identification of disease-associated genes that are enriched in non-neuronal cell populations, such as microglia and cerebrovascular cell types [13, 14].

In recent years, the cerebrovasculature and its role in the pathophysiology of neurodegenerative diseases has become a topic of considerable research focus. Despite extensive clinical data documenting functional changes in cerebrovascular function, especially at early stages of disease, the precise cellular and molecular mechanisms underlying these changes, particularly in humans, are not fully understood. Common themes of cerebrovasculature dysfunction, such as aberrant angiogenesis, decreases in tight junction expression, and increased transcytosis, have been noted, but the exact contributions of specific cerebrovascular cell types, and more importantly, their causal contribution to disease mechanisms are unknown. However, with the advent of a single cell atlas of the mouse cerebrovasculature, a framework for studying the molecular profiles of cerebrovascular cell types has been recently established [15]. This work provided a reference for several subsequent human atlases and the corresponding molecular changes within these cell types that occur across development and disease [14, 16–20].

This review focuses on our current understanding of cerebrovasculature structure and function and its role in neurodegenerative diseases. First, the cellular composition of the cerebrovasculature is described to highlight its unique structure with respect to the rest of the circulatory system and in turn, the specialized properties that it possesses. Second, a detailed review of molecular features is provided, with emphasis on recent single cell profiling studies that have revealed important differences between mice and humans. An overview of disease mechanisms across neurodegenerative diseases is then discussed to provide a framework for understanding recent efforts to elucidate the basis of enhanced vulnerabilities in disease. In particular, neuronal vulnerabilities within the common neurodegenerative diseases are examined as well as cerebrovascular cell type vulnerabilities within rare, inherited disorders. An overview into common themes of cerebrovascular dysfunction as shown by clinical measures as well as recent molecular profiling studies is then provided to highlight the contributing role of these changes in the progression of neurodegeneration. In turn, some of the most promising therapeutic approaches that emphasize the importance of considering the cerebrovasculature and other understudied brain barriers in drug development are presented. The review finishes by posing immediate outstanding questions in the field that have arisen from the latest research in the field and an outlook on how furthering our understanding of the cerebrovasculature will impact the field of neurodegeneration.

## Cellular and molecular architecture

### Cellular macrostructure

Broadly, the cellular composition of blood vessels can be phenotypically defined by two main descriptors - the specific segment or “zone” along the arteriovenous axis (e.g. artery, capillary, or vein) and the tissue parenchyma within which the vessels reside (e.g. lung, heart, kidney, brain) [21, 22]. At a fundamental level, zone function is identical across all organs, with arteries carrying blood away from the heart, capillaries facilitating transport activity between blood and tissue, and veins carrying blood to the heart. However, the physiological demands of each organ dictate the molecular phenotypes of vascular beds. Organotypicity of blood vessels therefore arises as a consequence of the unique cellular and molecular interactions between vascular and parenchymal cells rather than from an intrinsic property of vascular cells alone.

**Fig. 1:**
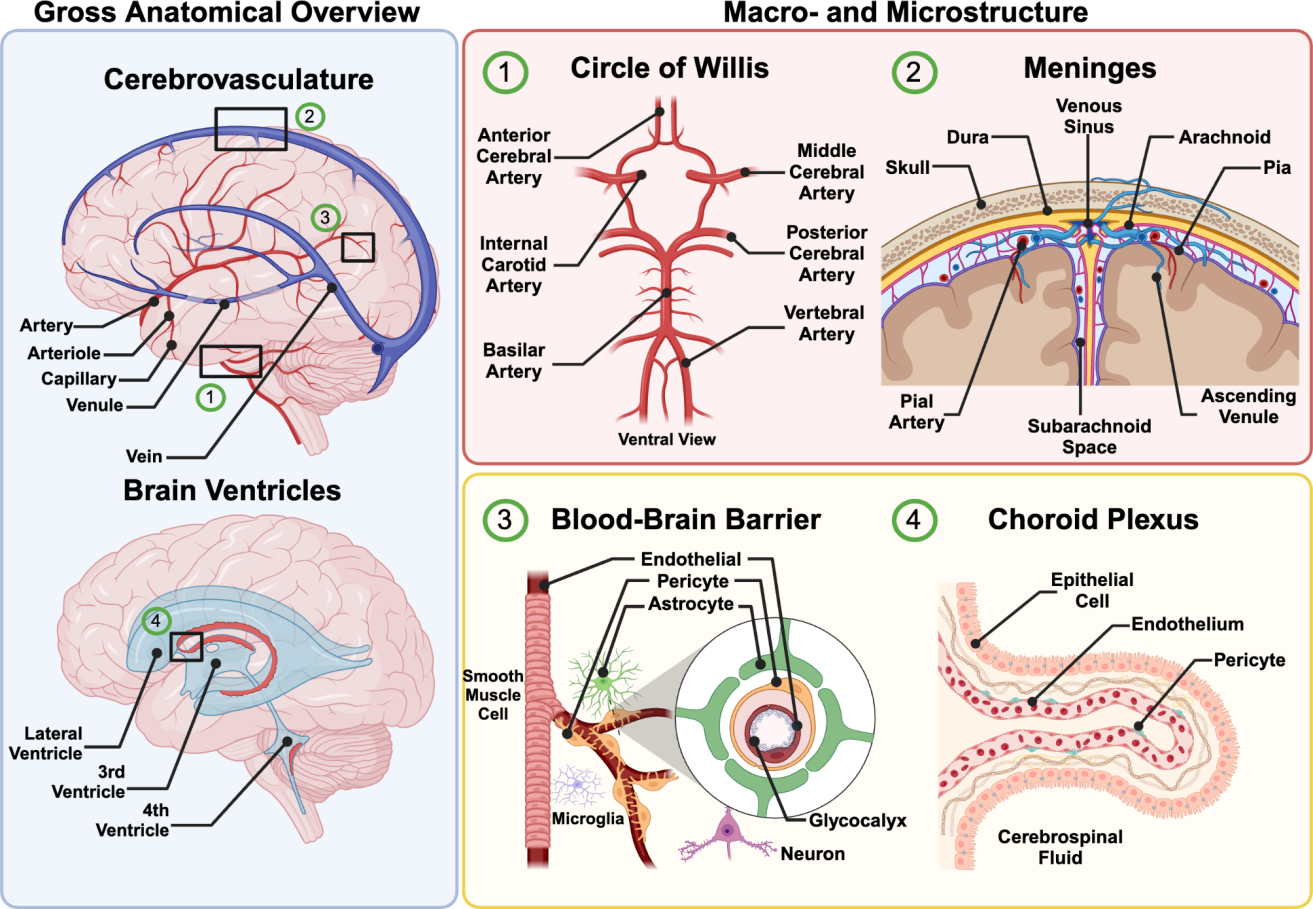
Anatomical overview of vascularization in the brain. Depiction of major structures comprising the cerebrovasculature and related structures in the brain. Gross anatomical overview (blue) highlights broad vessel types and brain ventricles. Insets (black rectangles) described in detail, grouped by larger macro- (red) and micro- (yellow) structures. Created in BioRender. https://BioRender.com/j83j024

The cellular and molecular composition of the cerebrovasculature follows the same principles as the vasculature in every other major organ but displays some of the most specialized properties in comparison (Fig. 1; Table 1). The brain is a highly metabolically demanding organ, utilizing approximately 20% of the body’s energy despite only constituting about 2% of the total mass [23, 24]. Given the lack of energy reservoirs in the brain, the cerebrovasculature must constantly provide high levels of oxygen, glucose, and other essential nutrients to sustain the function of neurons and glia. Secondly, not only is neuronal activity an energetically costly process, but changes to neuronal activity must be met with great spatial and temporal accuracy. Neurovascular coupling (NVC) refers to the close relationship between the cerebrovasculature and neurons, whereby local changes in cerebral blood flow are tightly regulated to sustain the metabolic demands posed by neuronal activity. Lastly, the cerebrovasculature is responsible for protecting vulnerable, non-renewable neurons from many insults, be it removal of toxic waste products or restricting the entry of pathogens and xenobiotics. This last specialization, more commonly referred to as the blood-brain barrier (BBB), is unique in that it restricts both paracellular and transcellular transport via the presence of tight junctions and suppressors of transcytosis, respectively [25, 26]. Given this, the cerebrovasculature relies on a vast assortment of transporters to regulate the influx and efflux of molecules from the brain parenchyma. In order to tightly regulate transport mechanisms throughout the parenchyma, the BBB is predominantly located at the level of the capillary bed, which comprises about 85% of the total cerebrovasculature [23]. It is therefore unsurprising that one of the most prevalent vascular phenotypes that arises in neuropathological conditions is BBB dysfunction [24, 27]. It is important to note that the cerebrovasculature and its aforementioned properties herein refer to blood vessels that reside within the brain parenchyma. Blood vessels are also found in all layers of the meninges as well as within the choroid plexus; however, these vessels exhibit their own specialized properties and form parts of different brain barriers discussed more extensively in a later section. In addition, vessels within circumventricular organs (CVOs), though often considered to be part of the cerebrovasculature, are highly permeable capillaries and thus do not exhibit specialized BBB properties [28].

Pial arteries branch off major arteries originating from the Circle of Willis and migrate along the surface of the brain before sending their own branches into the brain parenchyma [29]. Pial vessels reside within the meninges and thus do not exhibit specialized cerebrovascular properties. Interestingly, as soon as these vessels penetrate the brain they acquire BBB properties. The mechanisms underlying this abrupt phenotypic change at the interface between the pia and the brain are not fully understood, but recent work has highlighted the importance of the pial basement membrane in restricting brain vessel penetrance. In particular, the matrix metalloproteinase Mmp25 was found to be required by endothelial tip cells to cleave pial basement membrane collagen IV α5/6 chains for brain penetrance in a Wnt-β-catenin dependent manner, the same signaling pathway required for central nervous system (CNS) angiogenesis and induction of BBB properties [30, 31]. Whether this is a universal mechanism for BBB-competent vessel penetrance across organisms or even within all pial surfaces, however, remains an open question. Previous studies have demonstrated that the anterior and posterior meninges originate from different germ layers [32]. Consistent with this observation, recent molecular profiling of the meninges during development identified regional gene expression differences [33]. In particular, µ-crystallin (*Crym*) and *Serpine2* showed high expression within a particular pial subpopulation. Given that knockout of *Serpine2* is known to lead to hypervascularization in vivo [34], this raises the possibility that vascular patterning mechanisms could be variable across brain regions.

Vascularization of the brain is non-uniform. Topological patterning is distinct across brain regions and capillary densities can vary up to an order of magnitude [35]. For example, cortical regions exhibit alternating patterns of penetrating arterioles and ascending venules with a great degree of anastomosis. In contrast, the striatum is less vascularized with penetrating arteries occupying functional compartments and few anastomotic vessels [36]. The lenticulostriate arteries originating from the middle cerebral artery feed into the sensorimotor and associative compartments, whereas the recurrent artery of Heubner originating from the anterior cerebral artery feeds into the limbic compartment, thereby compartmentalizing the vascular supply of the striatum into two major territories (dorsal and ventral) based on the originating major cerebral artery. Despite this degree of heterogeneity, vascular patterning is stereotypic, suggesting a molecular basis exists for defining these patterns. Many studies have highlighted the importance of neuronal guidance cues during development on vascular patterning [37, 38]. However, how these signals differ across brain regions and how distinct neuronal and glial populations contribute to establishing the distinct patterning of the cerebrovasculature is not currently known. Cell type-specific profiling approaches likely will provide important insights into the mechanistic underpinnings of vascularization heterogeneity.

**Table 1:** Description of cerebrovascular and related structures

Structures	Description
**Circle of Willis**	**The network of arteries on the ventral side of the brain that provides most of the blood supply to the brain**
Anterior Cerebral Arteries	Major cerebral arteries branching off the Circle of Willis that provide blood supply to most midline regions of the frontal and medial parietal lobes
Middle Cerebral Arteries	Major cerebral arteries branching off the internal carotid arteries that provide blood supply to most lateral cortical regions, anterior temporal lobes, and insular cortices
Posterior Cerebral Arteries	Major cerebral arteries branching off the basilar artery that provide blood supply to the occipital lobe and medial and inferior temporal lobe
Lenticulostriate Arteries	Small arteries branching off the M1 segment of the middle cerebral artery that provide blood supply to parts of the basal ganglia, including the sensorimotor and associative regions of the striatum
Recurrent artery of Heubner	Small artery branching off the anterior cerebral artery that provides supply to parts of the basal ganglia, including the limbic region of the striatum
**Meninges**	**A collection of membranes that create a protective barrier surrounding the brain and spinal cord**
Dura Mater	The outermost layer of the meninges composed of thick fibrous tissue and supports the venous sinuses
Arachnoid Mater	The middle layer of the meninges composed of thin fibrous tissue and forms the arachnoid barrier with its outer layer
Pia Mater	The innermost layer of the meninges composed of a very thin layer composed of fibrous tissue that adheres to the surface of the brain and spinal cord
Pial Arteries	Arteries that pattern the surface of the brain on the pial surface prior to penetrating into the brain parenchyma
Subarachnoid Space	The space between the arachnoid and pia maters filled with cerebrospinal fluid
**Blood Brain Barrier (BBB)**	**The highly specialized border formed by brain endothelial cells that regulates the transport of molecules between the blood circulation and brain parenchyma**
Brain Endothelium	The cells that comprise the inner lining of blood vessels and exhibit selective and restrictive properties, including tight junctions, low rates of transcytosis, and specialized transporters
Mural Cells	The support cells on the abluminal side of the endothelium and includes smooth muscle cells and pericytes, essential for regulating blood flow and formation of the blood-brain barrier
Astrocytes	The major glial cell of the brain that extends processes onto the abluminal surface of the endothelium, forming end-feet that participate in ion and water homeostasis as well as neurovascular coupling
Glycocalyx	The layer of glycolipids, glycoproteins, and proteoglycans that cover the luminal surface of the endothelium and the provide first barrier for vascular transport across the endothelium
**Choroid Plexus**	**A vascularized structure formed by specialized epithelial cells within the cerebral ventricles that is responsible for the production and secretion of cerebrospinal fluid**
Cerebrospinal Fluid (CSF)	An ultrafiltrate of blood plasma produced by the choroid plexus and secreted into the cerebral ventricles and circulates around the brain providing a protective cushion
Ventricles	Interconnected cavities within the brain filled with cerebrospinal fluid, forming a ventricular system that bathes the brain
Epithelial Cells	Specialized cells that line the choroid plexus, forming the blood-CSF barrier, and are responsible for the production and secretion of CSF into the ventricles
Choroid Plexus Endothelium	The cells that comprise the inner lining of fenestrated blood vessels in the choroid plexus, distinct from BBB endothelial cells in that they do not exhibit similar barrier properties

### Cellular microstructure

Traditionally, cellular and molecular investigations of the cerebrovasculature have focused on the three main cell types that comprise the BBB: endothelial cells, pericytes, and astrocytes [26]. Brain endothelial cells that comprise the walls of blood vessels exhibit specialized barrier properties and serve as the interface between the brain and periphery. Pericytes, localized on the abluminal surface of capillaries, line the basement membrane and have been extensively studied for their roles not only in BBB development [39] but also their physiological roles in the mature brain. Being the analogous cell type to smooth muscle cells (SMCs) on larger vessels, pericytes and SMCs (collectively referred to as mural cells) share much of their molecular machinery and yet differ greatly in morphology [40]. Furthermore, the role of pericytes in regulating cerebral blood flow and relatedly the nomenclature for distinguishing pericyte subpopulations is a topic of great debate [41]. Astrocytes, in the context of cerebrovascular function, have been primarily studied for their roles in BBB maintenance, primarily through their end-feet contacts, as well as mediators of neurovascular coupling (NVC) [42]. More recently, their role in waste clearance has also become a topic of interest with the study of the glymphatic system and the elucidation of the role of aquaporin-4 function at astrocytic end-feet in mediating glymphatic flow [43]. Unlike mural cells and astrocytes, perivascular fibroblasts (PVFs) have not been as extensively studied but reside within the basement membrane, providing them direct contact with the vasculature. Although it is known that PVFs contribute extensively to the extracellular matrix (ECM), the diversity of PVF subpopulations and their precise functional roles is not fully understood [44–46]. Similarly less studied have been resident brain immune cell populations in the perivascular space of arterioles and venules, known as perivascular macrophages (PVMs) [47]. These cells are distinguishable from other myeloid cells in the brain, such as microglia and other border associated macrophages (BAMs), by both their anatomical location on parenchymal vessels (as opposed to within the meninges or choroid plexus) and expression of specific marker genes, such as *MRC1* (CD206) and *PTPRC* (CD45) [48–51]. Most recently, there is growing interest in understanding how PVMs and other BAMs might impact glymphatic flow [52] and neurovascular dysfunction [53] (e.g. through clearance of amyloid-beta), as well as their impact on haemodynamics, pathogen invasion, hypertension, stroke, subarachnoid hemorrhaging, and BBB permeability [54–57].

With the exception of PVFs and PVMs, all other brain cell types reside within the parenchyma and are separated by the near-complete coverage of the cerebrovasculature by astrocytic end-feet [58]. However, in recent years, direct interactions between the vasculature and other cell types as well as the mechanisms that mediate these interactions have become better understood. In particular, juxtavascular- or capillary associated-microglia have been shown to form direct contacts with the vasculature in regions devoid of astrocytic end-feet coverage and in part mediated by PANX1-P2RY12 signaling [59, 60]. Furthermore, oligodendrocytes have been shown to make direct contacts with the basement membrane in the neocortex, hippocampus, and cerebellar cortex [61]. Interestingly, synaptic-like transmissions were recently found between neurons and the vasculature through the action of glutamate NMDA receptor expression on arteriole SMCs [62]. This finding is particularly intriguing given recent findings that demonstrate caveolae vesicles on arteriole endothelial cells as important mediators of NVC [63]. Collectively, these studies highlight the importance of further characterizing the diversity of cell types and subtypes that are in proximity of the cerebrovasculature in order to understand cell type-specific mechanisms of cerebrovascular function.

### Molecular profiles

The broad implementation of next generation sequencing (NGS) based approaches to study brain cell types has greatly increased our understanding of the molecular basis for cellular identity. Even before the advent of single cell technologies, bulk RNA sequencing methods had provided invaluable insights into the transcriptomic profiles of cerebrovascular cell types. The use of fluorescent reporter lines has greatly aided in the efforts to purify specific cerebrovascular populations, thereby enabling the differentiation of transcriptomes from distinct cell types [64]. High resolution profiling of cerebrovascular translational programs has also been made possible with ribosomal tagging methods such as Translating Ribosome Affinity Purification (TRAP) [65, 66]. TRAP profiling of endothelial cells in development has allowed for the identification of organotypic factors in the brain, such as *Foxf2* and *Zic3*, which induce the expression of BBB-related genes [67]. However, the lack of single cell resolution has prevented, until recently, an understanding of the full breadth of cerebrovascular cell type heterogeneity in the mammalian brain.

The first single cell atlas of the mammalian cerebrovasculature was a foundational atlas for studying the molecular basis of cerebrovascular cell type composition and function [15]. By utilizing similar fluorescent reporter lines similar to those that previous studies had used for isolating specific cerebrovascular cell types, Vanlandewjick et al. were able to generate single cell transcriptional profiles for endothelial cells, pericytes, and PVFs from the mouse brain. This study uncovered that much like the phenotypic gradient that exists along the arteriovenous axis, gene expression within brain endothelial cells exists across a transcriptional gradient. For example, major facilitator superfamily domain-containing protein 2a (*Mfsd2a*), which has been identified as an essential gene for suppression of caveolae-mediated transcytosis at the BBB [68, 69], was shown to exhibit zonated expression at the capillary bed. In contrast, mural cell gene expression gradients did not appear to follow the anatomical organization along the arteriovenous axis. Instead, two distinct patterns were observed across the identified subpopulations of mural cells. Lastly, two subpopulations of PVFs were identified and localized to predominantly larger vessels (i.e. arterioles and venules) but not capillaries. These high resolution molecular profiles continue to provide invaluable insights into further understanding cell type-specific mechanisms in cerebrovascular function. Furthermore, this study has provided a reference for more recent work aimed at studying equivalent cerebrovascular profiles in the human brain.

The difficulty in constructing an equivalent cerebrovasculature atlas of the human brain came from the lack of adequate purification methods that did not rely on fluorescent labeling. To this day, most brain single cell atlases lack cerebrovascular cell populations or contain only a small pool of them in bulk profiling. Several studies in the past few years have developed enrichment strategies for capturing these cells from a combination of tissue sources, including fresh surgical resections of pediatric and adult patients, pre-natal and adult post-mortem tissue, and also from donors afflicted with a variety of neurological disorders [14, 16–19]. Many of the key findings for mouse cerebrovascular cell types were shown to be conserved in humans, including zonation of genes across the arteriovenous axis in endothelial cells and distinct patterns of mural cell gene expression that do not correspond to the anatomical axis. However, across all studies, while core functional roles for cell types were shown to be conserved across species, many human-specific differences were revealed at the molecular level. For example, novel marker genes *ARL15*,* TSHZ2*, and *ANO2* were found to be highly enriched in human brain arterioles, venules, and all endothelial cells respectively [16]. In contrast, genes previously shown to be important for restricting transcytosis at the BBB (*Vtn* in pericytes) or essential for transport of recently developed adeno-associated virus (AAV) capsids (*Ly6a* in brain endothelial cells) were shown to be specific to mouse cerebrovascular cells [70, 71]. Furthermore, fibroblasts have now been more thoroughly profiled in humans, with *KCNMA1* and *FBLN1* distinguishing at least two distinct subtypes [14, 16]. The precise spatial location and function of these molecularly specialized subtypes in humans is yet to be determined. However, recent single cell research profiling brain fibroblasts in mice has begun to match known anatomical positioning of brain fibroblasts with their respective molecular profiles [72]. Overall, the involvement of these genes in various processes of cerebrovascular biology (cell signaling - *ARL15*, transcription factor - *TSHZ2*, transporter activity - *ANO2*,* Ly6a* and *KCNMA1*, extracellular matrix composition - *Vtn* and *FBLN1*) highlights the importance of identifying and mapping species-specific differences within specific cell types.

Altogether, with the advances in single cell technologies and molecular approaches for capturing cerebrovascular cell populations, it is now possible to thoroughly interrogate distinct gene expression patterns with high resolution not only in animal models but in human samples as well. Unsurprisingly, core molecular characteristics, and in turn their functional consequences, are evolutionarily conserved. However, the precise molecular composition is distinct across species and divergences in gene expression are being shown to be essential for cerebrovascular function, thus highlighting the need to study characteristics of cerebrovascular biology in both animal models and humans.

## Enhanced vulnerabilities in neurodegenerative diseases

### Neuronal cell type vulnerabilities

**Fig. 2:**
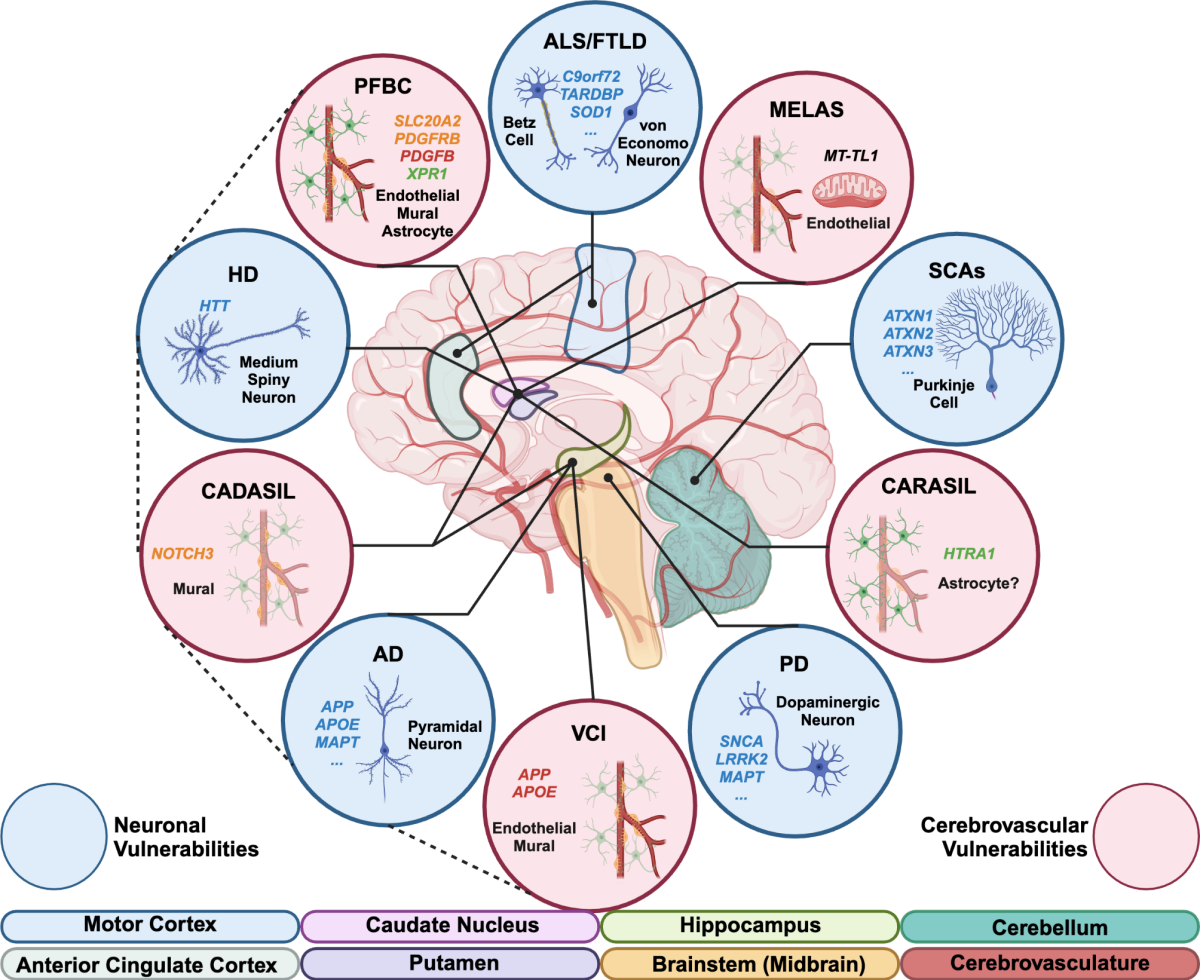
Cell type vulnerabilities in neurodegeneration. Implicated brain regions, cell types, and genes across common and rare, inherited neurodegenerative diseases are shown. Diseases are grouped by notably affected cell types (neuronal: blue; vascular: red). Dashed line: considerable overlap between linked diseases in terms of clinical manifestations and affected regions. Created in BioRender. https://BioRender.com/q21l434

Aging-associated neurodegenerative diseases encompass brain disorders that are marked by the progressive loss of select neuronal populations (Fig. 2). Alzheimer’s disease (AD), Parkinson’s disease (PD), Huntington’s disease (HD), spinocerebellar ataxias (SCAs), amyotrophic lateral sclerosis (ALS), and frontotemporal lobar degeneration (FTLD) are among the most common age-associated neurodegenerative diseases. Despite different clinical manifestations, these neurodegenerative diseases share many characteristics associated with disease progression [73] including mitochondrial dysfunction, disruption of proteostasis, and transcriptional dysregulation [74]. Single cell studies have recently highlighted that normal aging processes are heterogenous not only across brain regions but also across cell types [75, 76]. Unlike normal aging, however, neurodegenerative diseases are distinguishable by pathological hallmarks such as the aggregation of disease-linked proteins (e.g. amyloid-beta in Alzheimer’s disease) and selective neuronal cell death in affected brain regions (e.g. medium spiny neurons of the striatum in Huntington’s disease). With the exception of purely monogenic diseases such as HD and SCAs, the etiology for the majority of these neurodegenerative diseases is complex as many genes have been implicated through several genome-wide association studies (GWAS). Interestingly, disease-associated genes, including huntingtin (*HTT)* in the case of HD, are widely expressed across the brain and in many different cell types. This observation raises an interesting paradox whereby neurodegenerative diseases exhibit distinct patterns of enhanced neuronal loss and dysfunction in specific regions, but yet the genes most closely implicated in each disease are not restricted in expression to those corresponding regions. The phenomenon of enhanced cell vulnerability in neurodegenerative diseases remains one of the most intriguing unanswered questions in the field.

The polyglutamine diseases, including HD and the SCAs, represent a class of neurodegenerative diseases caused by trinucleotide CAG repeat expansions in particular genes [77, 78]. The mutated genes lead to progressive neurodegeneration of specific neuronal populations within the striatum (medium spiny neurons, MSNs) and cerebellum (Purkinje cells) of HD and SCA individuals, respectively. Given the affected brain regions, motor impairments are the most common symptom in these diseases, although as with most neurodegenerative diseases, as disease progresses, cognitive symptoms also develop. Most notable of these polyglutamine expansion disorders is that the degree of CAG repeat expansion in each disease gene inversely correlates with age of disease onset. A current hypothesis supported by recent GWAS for age-of-onset modifiers in HD posits that somatic instability in the CAG tract within vulnerable cells leads to expansions with age, until a pathogenic CAG length threshold is reached, at which point downstream gain-of-function toxicity effects lead to neurodegeneration in cells susceptible to the gain-of-function toxicity [79, 80]. The higher expression of some age-of-onset modifiers, such as *MSH3* (linked to CAG repeat instability [79]), in MSNs may provide insights into their specific vulnerability in HD despite the ubiquitous expression of *HTT* across many cell types. For this reason, efforts in drug development have expanded from HTT-lowering strategies to targeting some of these identified age-of-onset modifiers that have the potential of slowing or halting somatic CAG repeat instability [81], and thus affect disease progression. In the context of SCAs, although somatic instability has been studied and age-of-onset similarly inversely correlates with CAG expansion, no equivalent GWAS has to date been performed to identify age-of-onset modifiers [82, 83]. Furthermore, whether CAG expansions correspondingly occur within the vulnerable cell types such as Purkinje cells in SCAs is still an on-going area of research. Nevertheless, as with HD, genes linked to the SCAs are similarly broadly expressed across neuronal cell types, demonstrating the need to not only understand other drivers of disease but to further understand the individual roles of these mutated genes within specific cell types, both neuronal and non-neuronal.

Genetic forms of ALS and FTLD are most commonly caused by hexanucleotide (G_4_C_2_) repeat expansions in the *C9orf72* gene [84, 85]. Given the large overlap in clinical manifestations and pathological characteristics, particularly accumulation of transactive response DNA binding protein of 43 kDa (TDP-43), ALS and FTLD are often considered to lie on a disease spectrum [86]. Upper motor neurons in the motor cortex and lower motor neurons in the spinal cord are particularly vulnerable in ALS, whereas von Economo neurons (VENs) in the anterior cingulate cortex and frontal insula are those affected primarily in FTLD [87, 88]. Recent human transcriptomic studies using snRNA-seq have demonstrated that vulnerable cortical layer 5 neuronal populations in these regions exhibit indistinguishable gene expression patterns, reinforcing the previously observed overlaps across diseases and identifying a molecular basis for shared phenotypes [10]. Furthermore, based on transcriptional dysregulation patterns for all profiled cell populations and identified single-nucleotide polymorphisms (SNPs) from recent ALS GWAS studies [89], this study generated a predicted susceptibility score for differential vulnerability to demonstrate that cortical layer 5 cells in ALS exhibit the highest enrichment and thus co-expression of GWAS-identified genes. Given the lack of well-powered GWAS for FTLD, however, equivalent analyses with snRNA-seq data are currently not possible. Nevertheless, identification of novel associated genes in ALS/FTLD, including *TBK1*,* CCNF*,* and TIA1*, in addition to already known associated-genes in both diseases (*SOD1*,* C9orf72*,* TARDBP*,* MAPT*,* GRN*), demonstrates a growing overlap in pathogenic mechanisms underlying both diseases [90].

As the second most common neurodegenerative disease, PD has been extensively characterized in both its regional and cellular pathological patterns as well as underlying genetic factors contributing to disease [91, 92]. Dopaminergic (DA) neurons of the substantia nigra pars compacta (SNpc) are the most affected in PD despite similar DA neurons residing in the adjacent ventral tegmental area being relatively spared from degeneration [93, 94], suggesting specific characteristics render distinct DA populations more or less vulnerable to PD. Recent snRNA-seq profiling of post-mortem human tissue from individuals with PD identified distinct subpopulations of DA neurons within the SNpc, and highlighted a single subtype expressing angiotensin II receptor type 1, *AGTR1*, as being particularly vulnerable [12]. Furthermore, this subpopulation, and in general all SNpc DA neurons, were particularly enriched for disease-associated genes [92], including *SNCA*,* MAPT*,* NSF*, and *KANSL1*. Interestingly, only the *AGTR1*-expressing subpopulation was enriched for the disease-associated genes *ARL17B*,* WNT3*,* IGSF9B*,* ARHGAP27*,* KLHL7* and *PLEKHM1*, further highlighting its particular vulnerability. More recent profiling of PD SNpc using snRNA-seq has also highlighted a distinct neuronal population expressing the PD risk gene *RIT2* as being particularly vulnerable as well [95].

AD is the most common neurodegenerative disease and is pathologically characterized by the presence of amyloid-beta plaques and neurofibrillary, hyperphosphorylated tau tangles. Cognitive impairment and dementia are hallmark clinical manifestations, implicating regions involved in memory, such as the entorhinal cortex, CA1 of the hippocampus, and subiculum, as those most affected in AD [96–98]. These same regions are also involved in cognitive decline with normal aging. Recent progress in characterizing cell type-specific transcriptional changes in AD has shed important insights into the molecular basis for some of these observed vulnerabilities [8, 9]. For example, one subpopulation of CA1 excitatory pyramidal neurons and four subpopulations of entorhinal-specific excitatory neurons were found to be particularly depleted in AD individuals. Interestingly, when comparing expression of disease-associated genes from recent GWAS [99, 100], while neuronal populations did exhibit some level of enrichment, a large fraction of genes were enriched in non-neuronal cell types, for example 30 genes in microglia. Interestingly, additional enrichments were observed within cerebrovascular cell populations [14, 101], which is further discussed in a later section. This overlap of AD with cerebrovascular pathology is intriguing given that they often co-exist together, and vascular dementia accounts for about 20% of all cases of dementia [102]. The terms vascular cognitive impairment (VCI) and dementia (VCID) are now used to define conditions in which vascular injury underlies cognitive impairments and decline [103], encompassing uniquely vascular insults such as arterial occlusions [104], mixed AD and vascular pathologies such as cerebral amyloid angiopathy (CAA) [105], and purely genetic causes such as cerebral autosomal dominant arteriopathy with subcortical infarcts and leukoencephalopathy (CADASIL) [106]. By definition, the vulnerable cell populations in VCI/VCID are those that comprise the cerebrovasculature, namely, endothelial cells, mural cells, and perivascular cells such as PVFs and PVMs. As shown with molecular profiling studies in AD, mixed cerebrovascular and neuronal signatures of cell type vulnerability arise in analyses, thus making it difficult to ascertain key drivers of disease.

With recent advances in human molecular profiling and higher-powered genetic studies, the underlying basis for the phenomenon of enhanced vulnerabilities is becoming increasingly clearer. Enrichment of disease-associated genes or modifiers of disease within vulnerable cell populations is providing key insights into the mechanisms that drive neurodegeneration. Interestingly, despite robust signatures of vulnerability within the extensively studied affected neuronal populations, identification of risk genes that are enriched within non-neuronal populations is also providing a novel perspective for understanding the mechanisms underlying neurodegeneration. As seen across the neurodegenerative diseases, most highly implicated genes, even in purely genetic forms, are not restricted in expression to the most affected neuronal populations. In this light, future studies focusing on cell type-specific contributions of disease-associated genes are likely to disentangle the precise roles and contributions in disease pathology.

### Direct genetic links between vascular dysfunction to neurodegeneration

The interconnections between many affected cell types and pathways often makes it difficult to ascertain what are the primary causal drivers in neurodegenerative diseases. Some of the earliest cellular and molecular changes that occur in disease are not exclusively found in neurons. Cerebrovascular dysfunction often precedes the onset of more overt neurological features, but the extent to which this causally contributes to disease progression is not fully understood [24, 27]. Even so, evidence from many rare neurological disorders demonstrates that alterations to genes primarily expressed by cerebrovascular cells can lead to neurodegeneration, and in some cases, phenocopy some of the clinical manifestations observed in the more common neurodegenerative diseases (Fig. 2).

CADASIL is a rare inherited disorder and the most common genetic cause of vascular dementia. CADASIL is linked to mutations in *NOTCH3* [106], a gene expressed primarily by smooth muscle cells, and mutations in this gene lead to accumulation of the NOTCH3 protein ectodomain in the extracellular spaces and consequently the formation of granular osmiophilic material. Over time, affected individuals develop seizures, cognitive and memory impairments, and eventually dementia. As with many cerebral small vessel diseases, CADASIL primarily affects white matter and basal ganglia regions, presenting with white matter hyperintensities and lacunar strokes on neuroimaging [107]. Interestingly, hippocampal atrophy from select loss of a subpopulation of pyramidal projection neurons in the hippocampal formation has been noted to likely be involved in the memory and cognitive impairment aspects of the disease [108, 109].

Similar to CADASIL, cerebral autosomal recessive arteriopathy with subcortical infarcts and leukoencephalopathy (CARASIL) is a genetic form of vascular dementia, caused by mutations in the *HTRA1* gene [110]. Much like CADASIL, CARASIL affects small penetrating arterioles in the white matter and basal ganglia but lacks the canonical granular osmiophilic material depositions in the extracellular spaces. Nevertheless, smooth muscle cell loss and thickening of the extracellular matrix is also present. At a molecular level, *HTRA1* mutations result in the loss of TGFβ signaling suppression, resulting in aberrant remodeling of the vasculature [111]. Although not much is known about the cell type-specific changes in CARASIL, single cell studies have demonstrated that *HTRA1* expression is predominantly found within astrocytes rather than endothelial or smooth muscle cells [4]. Even so, disease phenotypes originate in the cerebrovasculature and consequently lead to similar symptoms as CADASIL, including lacunar strokes, mood and cognitive impairments, and eventually dementia.

Primary familial brain calcification (PFBC), formerly known as Fahr’s disease, is a rare neurodegenerative disorder characterized by bilateral calcifications primarily in the basal ganglia [112]. Heterozygous variants in several genes, including *SLC20A2*,* PDGFRB*,* PDGFB*, and *XPR1* have been identified to cause autosomal dominant forms of the disease [113, 114]. Both *SLC20A2* and *PDGFRB* are primarily expressed by mural cells, specifically pericytes, whereas *PDGFB* and *XPR1* are expressed by endothelial cells and astrocytes, respectively. All genes are involved in phosphate transport, which accounts for the primary pathology of perivascular calcifications. Individuals with PFBC typically experience movement disorders, cognitive deficits, and psychiatric disturbances. Given the clinical presentations and the affected brain regions, PFBC in some aspects mimics aspects of Huntington’s disease, though isolated chorea is not present in PFBC.

Mitochondrial encephalopathy, lactic acidosis, and stroke-like episodes (MELAS) is a unique disease in that it exemplifies a paradox of disease biology - affecting a fundamental process of cell biology (i.e. mitochondrial function) and yet displaying specific pathological features [115]. The most common mutation underlying MELAS occurs from an adenine-to-guanine transition at position 3243 of the mitochondrial genome (m.3243 A > G), affecting a mitochondrially-encoded tRNA [116]. As with many mitochondrial diseases, dysfunctional production of ATP via the oxidative phosphorylation pathway is the main driver of disease, shifting metabolic activity into a chronic lactic acidosis state. This combined with seizure and stroke-like events are the characteristic diagnostic features for MELAS. Interestingly, recent studies suggest a vascular pathophysiological basis for the unique presentation of MELAS [115, 117, 118]. Angiopathy, present within cerebral arteries, arterioles, and capillaries, is a unique clinical feature which is absent from other mitochondrial diseases. In addition, basal ganglia calcifications are also observed, particularly once the disease progresses to chronic stages [119, 120]. Furthermore, enlarged mitochondria and abnormal respiratory chain mechanisms have been well-documented, supporting the involvement of vascular cell types in the pathogenesis of the disease [118]. Over time, affected individuals develop additional systemic and neurological symptoms including aphasia, migraines, and dementia.

Collectively, these disorders represent a handful of examples in which genetic defects acting within vascular cell types themselves drive neurodegeneration. The precise molecular mechanisms underlying each disease is unique, but the convergence in affected brain regions, clinical manifestations, and vascular pathologies provides an interesting framework for better understanding cell type-specific contributions of the cerebrovasculature to neurodegeneration. Notably, the basal ganglia, hippocampus, and white matter regions are commonly affected across these genetic disorders. Why pathology initiates within cerebrovascular compartments in these subcortical regions is not known, but interestingly, these regions exhibit some of the lowest densities of blood vessels [35], suggesting the genetic programs responsible for regional vascularization could underlie the observed vulnerabilities of these regions.

## Cerebrovascular dysfunction in neurodegeneration and aging

### Clinical manifestations of cerebrovascular dysfunction

As noted above, most of the common age-related neurodegenerative diseases have complex etiologies and many disease-associated genes have been implicated, including some which would be predicted to directly involve cerebrovascular function. Cerebrovascular dysfunction is now appreciated to occur across many of the neurodegenerative diseases and typically manifests at early stages in the disease progression [24, 27]. A mixture of clinical measurements, including cerebral blood flow measurements (CBF), transporter activity, and BBB permeability have been performed across the neurodegenerative diseases. Interestingly, similar patterns of cerebrovascular impairments have been identified that coincide with known regional vulnerabilities for each respective disease.

Studies in individuals with mild cognitive impairment and early AD have shown significant changes in CBF within several brain regions, including the hippocampus and parietal areas [121, 122]. *APOE4* carriers exhibit CBF reductions across frontal, temporal, and parietal cortices as well as corresponding reductions in glucose uptake [123]. Similarly, hypoperfusion in PD patients has been detected within posterior cortical regions and correlates with observed motor impairments and global cognitive performance [124, 125]. Interestingly, P-glycoprotein, the main efflux pump at the BBB encoded by *ABCB1*, shows decreased activity in affected cortical areas of AD as well as the midbrain in PD [126, 127]. Within ALS, using similar arterial spin labeling approaches, perfusion changes in frontal and parietal cortical regions were shown to be correlated with disease severity irrespective of brain atrophy [128, 129]. Analogously, CBF reductions have been detected at early stages of HD within the caudate nucleus and the putamen as well as hypometabolism of fluoro-deoxy-glucose [130–132]. Similar findings within white matter regions of MS patients have also been documented [133].

Functional measurements of BBB permeability using dynamic contrast-enhanced magnetic resonance imaging (DCE-MRI) have found similar patterns as with observed CBF and transporter activity changes. Notably, in both normal aging [134, 135] and AD [136] BBB permeability has been observed within regions of the hippocampus. Similar findings have been found within the substantia nigra of PD patients [137], caudate nucleus of HD patients [138], and white matter regions of MS patients [139]. As discussed in the next section, these observed increases in BBB permeability are consistent with molecular changes that manifest at early stages of disease within the affected brain regions of both human samples and mouse models.

### Common cellular and molecular alterations at the BBB

Much of what is known about cerebrovascular dysfunction at the cellular and molecular levels in neurodegeneration comes from a combination of animal model studies as well as post mortem human studies. Only recently have human single cell studies begun to emerge for specific neurodegenerative diseases [8–12], and even within these, a limited number have focused on cerebrovascular cell types [14, 16, 101]. However, to date it is known that key pathways involved in BBB structure and function as well as immune-related and angiogenic/vascular remodeling are dysregulated across diseases. Furthermore, as with clinical manifestations noted above, cellular and molecular changes occur at early stages in disease progression within vulnerable regions. The precise involvement of cerebrovascular cell populations (e.g. zonal differences, pericyte activation, end-feet polarization and gliosis) as well their interactions with vulnerable neuronal populations, however, are not well understood.

Cellular and molecular studies focusing on the cerebrovasculature of HD and SCAs have been limited. *HTT* is known to be widely expressed across many cell types, including those of the cerebrovasculature. In HD, mHTT protein has been found within cerebrovascular cell types, including endothelial cells, mural cells, and perivascular macrophages [138]. The fraction of detected mHTT produced by cerebrovascular cells versus that which may be in the process of being transported across the BBB as a clearance mechanism, however, has not been resolved. Furthermore, direct contributions of cerebrovascular-derived mHTT to disease progression are unclear. Post-mortem human as well as animal models studies have demonstrated dysfunction of the BBB within the striatum [16, 138, 140], namely based upon decreases in endothelial/BBB markers, such as *CLDN5*,* TJP1*,* SLC2A1*,* TFRC*, and *MFSD2A*. Functionally, this is consistent with observations in these studies of increased BBB permeability both via paracellular (i.e. decreased tight junction levels) and transcellular (i.e. increased vesicular trafficking) routes. Furthermore, an increase in small vessel density has also been observed in both HD animal models and humans, which may be attributable to aberrant activation of HIF1A-mediated Wnt signaling, as has been shown through differentiated induced pluripotent cells from HD donors [141]. Pericyte activation as well as innate immune signaling have also been shown to co-occur with the dysfunction of the endothelium in HD [16, 142]. Within the SCAs, the only mechanistic insights into cerebrovascular dysfunction come from work in an animal model and post-mortem analysis for SCA3, caused by CAG expansions in the *ATXN3* gene [143]. Analogous to HD, BBB dysfunction via extravasation assays of Evans Blue and fibrinogen were observed within the cerebellum of mice and human samples, respectively. In addition, aggregates of ATXN3 were localized to cerebellar blood vessels where extravasation was present, raising the same question, as in HD, of the degree of cerebrovascular-derived mutant gene expression and its cell autonomous role in cerebrovascular dysfunction.

Cerebrovascular dysfunction has also been noted in the motor cortex and spinal cord of both humans and animal models of ALS and to a lesser degree within FTLD. Whereas previous studies focused on the blood-cerebrospinal cord barrier (BCSFB) found downregulation of tight junction proteins and a reduction of pericyte numbers [144–147], recent transcriptional profiling of both the motor and prefrontal cortices of ALS and FTLD has shown cerebrovascular cell abnormalities, predicted to lead to BBB dysfunction, within the motor cortex, though more pronounced in ALS compared to FTLD [10]. Important insights from monogenic mouse models of ALS, particularly *SOD1* G93A mutants, have shown that cerebrovascular dysfunction precedes the onset of motor neuron degeneration [148–151]. Additional studies have focused on the causal role of ALS/FTLD-associated gene *TARDBP* in vascular dysfunction. Conditional knockout of TDP-43 in both mice and zebrafish leads to disruption of tight junction proteins and vascular patterning abnormalities [152, 153]. Interestingly, TDP-43 pathology has been demonstrated in ALS/FTLD patients both within the basal lamina as well as within vascular cells [154, 155], inspiring work into understanding the function of TDP-43 within cerebrovascular cell types. Post-developmental deletion of endothelial TDP-43 leads to defects in vascular sprouting and barrier defects of the retinal vasculature as well as BBB disruption via reduction of Wnt signaling and increased inflammation from microglia and astrocytes [156]. Similar findings were observed in brain endothelial-specific deletion of *Tardbp* and *Grn* mice which displayed fibrin deposition consequential to BBB disruption as well as activation of glial cell populations. Interestingly, this work also demonstrated a behavioral consequence of brain endothelial-specific deletion, highlighting the direct causal disease role of cerebrovascular function [157].

Similar to HD and ALS/FTLD, studies focusing on cerebrovascular dysfunction in PD recapitulate the findings of BBB disruption and aberrant angiogenic/vascular remodeling phenotypes [158, 159]. Human and animal models have noted these changes to occur within basal ganglia structures, similar to HD, with the exception that in PD these changes also occur within the substantia nigra where the most vulnerable dopaminergic neurons are located. Increased capillary density within the substantia nigra, particularly “string vessels” with collapsed basement membranes and no functional perfusion, have been identified in both non-human primate models and PD patients [160, 161]. These studies are consistent with a notable reduction of glucose metabolism in the substantia nigra as observed through [^18^F]-fluorodeoxyglucose PET imaging [162]. Furthermore, reduced function of the major efflux pump, P-glycoprotein (*ABCB1*), via [^11^C]-verapamil PET imaging has also been observed within the midbrain of PD individuals [127]. In relation to the genetics of PD, alpha-synuclein expression has been noted in vascular cell population, though preferentially in endothelial and smooth muscle cells of meningeal versus parenchymal vessels [163]. The precise role of vascular-derived alpha-synuclein in normal or pathological states, however, is not known. However, the higher expression in meningeal vessels is noteworthy given recent studies demonstrating the importance of PVMs in glymphatic clearance and observed impaired flow within the A53T mouse model of PD [47, 164]. In addition, BBB dysfunction both via downregulation of tight junction proteins and leakage assays have been shown within the A53T and alpha-synuclein models of PD [165, 166].

Representing 60–80% of all dementias, 45% of which are estimated to be related to small vessel diseases, AD accounts for a large amount of our understanding of cerebrovascular pathology in neurodegeneration [24, 27, 158]. As with other neurodegenerative diseases, dysregulation of tight junction protein, BBB leakage, and immune activation along with deposition of blood-derived proteins have been noted within affected prefrontal and entorhinal cortical regions and the hippocampus of AD brains [167–169]. Most recently, molecular profiling of cerebrovascular cells within AD brains has highlighted cell type-specific transcriptional dysregulation [14]. In particular, fewer numbers of AD cerebrovascular cells compared to controls were obtained in this study, consistent with known loss of pericytes and endothelial cells as well as the presence of “string vessels” in AD [170–172]. Furthermore, in this profiling study, a large proportion of top GWAS hits were identified to be enriched within specific cerebrovascular cell subpopulations, highlighting the close ties between cerebrovascular function and AD etiology. Mechanistically, studies in animal models of AD focusing on the cerebrovasculature have primarily focused on the role of amyloid-beta pathology, especially within the context of amyloid-beta clearance and the deposition of plaque on vessel walls, a condition known as cerebral amyloid angiopathy (CAA). For example, genetic risk factor *Picalm* and low-density lipoprotein receptor-related protein *Lrp1* have both been shown in vivo to be involved in transcytosis at the BBB [173–175]. Interestingly, *PICALM* and *LRP1* are enriched in SMCs and endothelial cells, respectively, in the human brain, suggesting cell type-specific mechanisms exist for the trafficking of amyloid-beta [14, 16]. Furthermore, *APP* itself, specifically the splice isoform APP700, has been shown to be expressed by brain endothelial cells in vitro to produce Aβ40 and Aβ42 [176]. This particular form of amyloid-beta has sialylated core 1 type O-glycans and is preferentially cleaved by both BACE1 and γ-secretase in brain endothelial cells. Given the enrichment of glyco-genes in brain endothelial cells for maintaining the glycocalyx [177], especially O-mucin type glycans, future work in animal models and humans will provide important insights into the interactions between the cerebrovascular glycocalyx and pathogenic mechanisms of aggregate clearance in neurodegeneration. In addition to these interactions, the interplay of CAA pathology, other cerebrovascular cell types, and genetic risk factors, such as *APOE*, is an ongoing field of research [178]. *Apoe* knockout mice exhibit compromised BBB function as assessed by extravasation of Evans Blue conjugated to albumin [179], demonstrating an essential role for APOE in maintenance of the BBB. However, *Apoe4/4* mice exhibit reduced capillary basement membrane surface area [180], and the APOE ε4 isoform is known to enhance cerebrovascular dysfunction and amyloid-beta pathology, including CAA [181, 182]. Furthermore, it has been observed that APOE and amyloid-beta both bind to LRP1 at the BBB [183]. Recent work has highlighted the role of APOE ε4 genotype and BAMs in the pathogenesis of CAA, noting these cells as a cellular source of reactive oxygen species and consequently, inducing neurovascular dysfunction [184, 185]. Collectively these works demonstrate the complex interactions between genetic risk factors for AD and cerebrovascular function in the pathophysiology of the disease. In contrast to amyloid-beta, studies focused on the role of neurofibrillary tau tangles on cerebrovascular pathology have found them to accumulate in brain endothelial cells of AD tau mouse models and humans [186, 187]. Tau overexpression in vivo leads to abnormal changes to blood vessels including increased densities and reduced diameters, accompanied by increased expression of angiogenesis-related genes such as *Serpine1* and *Vegfa* [188]. Interestingly, tau overexpression in astrocytes alone also leads to BBB disruption and extravasation of albumin protein [189]. Mechanistic studies have also determined that accumulation of soluble tau leads to blockage of endothelial nitric oxide synthase activation and consequently, neurovascular uncoupling [190, 191]. Collectively, these studies highlight the role of tau in mediating glial inflammatory responses that subsequently lead to BBB disruption [192].

Altogether, it is clear that common themes of cerebrovascular dysfunction are present across the neurodegenerative disease and typically manifest at early stages within the most vulnerable brain regions. Though this suggests that cerebrovascular dysfunction is secondary to the neuronal changes, the complex interactions, particularly with disease-associated genes, implies that cerebrovascular function is more involved in causal mechanisms of neurodegeneration than previously appreciated. At the very least, cerebrovascular dysfunction is likely to create a permissive environment for neurodegeneration, and thus presents itself as a promising target for the development of therapies without the need of crossing the BBB.

## Therapeutic insights and future directions

Over the past few decades, clinical trials for neurodegenerative disease modifying therapeutics have met with great failure. Since 2021, only three anti-amyloid antibodies have been approved by the Food and Drug Administration (FDA) for the treatment of AD, and in HD, all huntingtin-lowering approaches using antisense oligonucleotides have failed to meet clinical endpoints. Despite in each case highly compelling evidence from preclinical animal model studies, there is still a great discrepancy between preclinical and clinical outcomes. In part, a full understanding of the principles governing molecular and cellular principles at transport interfaces in humans, namely the brain barriers, and adequate delivery strategies to cross these barriers has been lacking. In light of recent advances within the fields of single cell genomics (documentation of molecular profiles for many understudied rare cell types of the brain) and gene therapy (development of improved tools for crossing the BBB), many promising approaches for treating neurodegenerative disease are now being investigated.

### Brain barriers: the choroid plexus, meninges, and glycocalyx

The cerebrovasculature represents one of several major interfaces between the blood and the brain. Although not as extensively studied as the cerebrovasculature, the choroid plexus and the meninges also both provide essential roles in supporting brain function. In recent years, studies focusing on each of these barriers have now extensively characterized the diversity of cell type composition and function as well as their roles in diseased states. In addition, the importance of the glycocalyx as the first interface with transport mechanisms at the BBB and its role in neurodegenerative diseases is becoming increasingly understood. Although much is still unknown regarding the mechanisms underlying their functions, it is clear that these structures and their relevance to neurodegenerative diseases is more important than previously appreciated.

The blood-cerebrospinal fluid (CSF) barrier is provided by the choroid plexus, a structure composed of specialized epithelial cells that secrete CSF into the ventricles that fills the subarachnoid space surrounding the brain. Humans produce between 400-600mL of CSF a day, which allows the brain to exist in a buoyant state for protection from physical injuries and also clear waste products. The rich composition of CSF biomolecules has been extensively studied and thus the interest in accessing CSF both for identifying biomarkers of disease and as a drug delivery route have received considerable attention [193]. Recently, the interest in understanding molecular profiles of cell types that comprise the choroid plexus has been investigated [194]. Interestingly, distinct gene expression patterns were found within each of the choroid plexus regions, both for the epithelial cells as well as the embedded mesenchymal cells, consistent with prior work demonstrating differential developmental trajectories for each region [195]. Whether these gene expression patterns are conserved in human choroid plexus or whether additional specific-specific adaptations are present, however, remains unknown. Most recently, multi-modal approaches including TRAP have shown diurnal fluctuations in gene expression within the mouse choroid plexus including the highly expressed epithelial marker *Ttr* [196]. This work is consistent with the known roles of CSF in waste clearance and the sleep-wake cycle, as well as the activity-dependent ABC transporter diurnal fluctuations that have also been observed within brain endothelial cells and their dependence on clock gene machinery [197].

The dura, arachnoid, and pia mater, collectively referred to as the meninges, have historically been known as physical barriers that surround and protect the brain. While the anatomical composition of each part has been known for several decades [198], the underlying molecular basis and diversity of cellular composition has only recently been investigated across several studies [33, 44, 72]. The meninges harbor a wide repertoire of fibroblast subtypes across all three layers, each with distinct gene expression profiles. Furthermore, the arachnoid barrier cell layer forms a functional barrier within the meninges through the expression of junctional proteins including *Cldn11* and *Cdh1* (i.e. E-Cadherin). Lastly, the meninges, specifically within the dural sinuses, have been shown to harbor several populations of immune cells [199]. The precise functional role for each population of meningeal cells is still under investigation, both in homeostatic and pathological states. Of great interest recently has been the connection between CSF flow and interstitial fluid (ISF) within the brain through the glymphatic system [43]. As mentioned above, the subarachnoid space, located between the arachnoid and pia mater, is filled with CSF and flows along perivascular spaces surrounding pial vessels that penetrate into the brain. Through poorly understood molecular mechanisms, CSF flows along periarterial spaces driven by arterial pulsatility, intermixes with ISF partially mediated by the water channel aquaporin-4 (*Aqp4*), and drains along perivenous spaces. Together with the identification of dural lymphatic vessels that drain to cervical lymph nodes, the glymphatic system is thus connected with the peripheral lymphatic system [200]. The extent to which the glymphatic system enables proper waste clearance, particularly of amyloid beta, and its direct contribution to pathological conditions remains unclear [201]. More knowledge of these structures is essential for a better understanding of brain fluid dynamics, which are greatly affected in neurodegenerative diseases. Future experiments aimed at providing key insights into their governing molecular mechanisms will in turn aid in determining the feasibility of targeting this system for developing novel therapies.

Made from a diverse assortment of glycoproteins, proteoglycans, and glycosaminoglycans, the endothelial glycocalyx provides the first distinct barrier property between the blood and the parenchyma [202]. The role of the glycocalyx in the BBB has been poorly studied, but yet, it is known to exhibit distinct functional properties compared to other tissue glycocalyx [177, 203, 204]. In particular, lipopolysaccharide-induced injury of the entire vasculature has demonstrated that the cerebrovascular glycocalyx is more resistant to injury, suggesting it confers greater protective characteristics for the brain. With recent advances in molecular profiling of human brain endothelial cells, the repertoire of genes that regulate glycocalyx composition can now be extensively interrogated to further dissect the basis for these specializations in the brain.

The importance for understanding the underlying cellular and molecular mechanisms for these understudied structures is exemplified by recent insights into their disrupted functions during AD and aging [205–207]. Given the observed cerebrovascular changes across neurodegenerative diseases, it is likely that CSF, meningeal, and glycocalyx impairments such as disrupted glymphatic flow, waste clearance, and glycocalyx structure extends to these diseases as well. In fact, recent evidence within knockin models of HD have demonstrated that CSF clearance efficiency is impaired prior to the onset of motor symptoms and worsens with disease progression [208]. Future studies will provide invaluable information on cell type-specific roles in maintaining these barriers within normal and diseased contexts as well as the potential for targeting these structures with novel therapeutic approaches.

### Gene therapies and engineered transport technologies

With advances in our understanding of basic molecular and cellular profiles of disease mechanisms as well as improvements in drug chemistry and bioengineering, new translational programs have now begun to enter preclinical and clinical stages that have the potential of revolutionizing the field of neurodegenerative disease treatment. Many pharmaceutical programs are now devoting efforts to novel gene editing and drug delivery approaches, with the hope of developing therapies with better brain bioavailability, specificity, and therapeutic efficacy.

Antisense oligonucleotides (ASOs) based therapeutics, which are used for lowering mutant disease-linked gene mRNA and protein, remain one of the most promising approaches for gene targeting given their landmark successes in neuromuscular disorders such as spinal muscular atrophy and Duchenne muscular dystrophy [209]. However, recent major setbacks, specifically in applying ASOs for huntingtin lowering in HD, have prompted a refocus on alternative therapeutic approaches. This is in part due to the realization that ASOs administered through lumbar CSF lack good penetration into deep brain structures [210]. In diseases such as HD and PD, which affect deep subcortical regions, drug delivery of ASOs remains a limiting factor based on current delivery approaches. Nevertheless, improvements in ASO chemistry, administration paradigms, and the development of novel BBB transport strategies hold promise for future successes, especially given the widely validated preclinical benefits of lowering mutant protein levels in each disease context [211].

The utility of transport vehicles that rely on receptor-mediated transcytosis at the BBB has been explored for many years [212–214]. These “trojan horse” approaches take advantage of endogenous receptors on the BBB, such as those for transferrin, insulin, and lipoproteins, to shuttle therapeutic cargo across the endothelium. Recent advances in transport vehicle designs have focused on targeting of the human transferrin receptor (TfR) by engineering an Fc domain that can be combined with variable therapeutic moieties, such as Fabs, enzymes, and oligonucleotides [215, 216]. This modular approach is now being implemented for several neurological indications, including Hunter’s Syndrome and FTLD [216, 217]. One caveat, however, is the widespread expression of TfR across multiple other organs. The desire for brain-specific delivery has led to efforts to identify and characterize additional receptors (e.g. basigin, Glut1, and CD98hc) that could similarly be “hijacked” for drug delivery using similar transport vehicles [218, 219].

Similar to transport vehicles, adeno-associated viruses (AAVs) have also drawn considerable attention in recent years due to advances in directed evolution for capsids that can more readily target specific brain cell populations [220–222]. By screening for capsids that target any brain cell type or even subtype of interest, many researchers are now able to further interrogate cell type-specific disease mechanisms in animal models. Furthermore, these tools can be administered either directly into the brain or engineered to access the brain via intravenous delivery routes, thereby crossing the BBB using endogenous molecular machinery within the organism. Such non-invasive delivery is appealing on order to avoid the need for complicated surgical procedures such as intrathecal or intracerebral administration, but also has the drawback of cost (associated with the need to produce large quantities of virus) as well as potential toxicity from peripheral off-targeting or immune responses. For this reason, optimizing viral production to yield potent AAVs that can be used at lower titers as well as further understanding mechanisms governing AAV transport are currently topics of intense research. In particular, understanding the mechanisms of AAV transduction is crucial given the mechanism-agonistic approaches used for viral screening. Older generations of AAV9 variants (i.e. PHP.eB, PHP.S, and PHP.B) were evolved within common mouse strains with no understanding of the mechanism underlying their BBB transport. *Ly6a*, a gene expressed exclusively on the brain endothelium of certain mouse strains and absent entirely from non-human primates and humans, was later identified to be an obligate receptor for transport of these novel capsids [70, 223]. With this in mind, capsid evolution is now focused on identification of putative receptors that are known to be conserved across organisms or at least highly expressed in non-human primates and humans for clinical applications. With this approach, novel receptors such as LRP6 and CA4 have been identified as targets for newer generations of evolved AAV capsids [224, 225]. In combination with recently identified cis-regulatory regions from single cell assay for transposase-accessible chromatin sequencing (scATAC-seq) datasets of the human brain, future generations of evolved AAVs will greatly aid in proper targeting of affected brain cell types across neurodegenerative diseases [226–228]. Indeed, recent efforts to translate optimized AAVs into preclinical and clinical settings have also demonstrated great promise. In particular, improved AAVs for targeting medium spiny neurons and lowering huntingtin for Huntington’s disease treatment have recently emerged [229].

Altogether, these novel therapeutic modalities hold great promise for targeting neurodegenerative diseases at symptomatic and even presymptomatic clinical stages. Mechanistic insights from prior studies as well as a plethora of brain single cell studies provide strong premises for the validity of these approaches. With increased understanding of brain barrier molecular profiles across organisms, streamlined platforms for developing brain-penetrant therapeutics, and efficient identification of disease-modifying targets, efforts to treat and halt neurodegenerative diseases will likely meet with major success within the next few decades.

### Outstanding questions and future directions

The molecular profiles of cerebrovascular cell types across development, normal adulthood, and various pathological states are proving to be invaluable for not only understanding underlying disease mechanisms but also improving our design of new approaches for brain therapeutic delivery. Nevertheless, many knowledge gaps remain which, when answered, will provide essential insights into our understanding of brain function. Single cell genomics, cell type-specific targeting, and functional imaging approaches are key to bridging insights into basic cerebrovascular function and a mechanistic understanding of disease biology.

Cell type and subtype diversity can be classified under many criteria, whether it be transcriptional programs, morphology, or function. As observed with cerebrovascular cell types, fundamental functions are conserved across species and organ systems. Even so, important distinctions can be identified which often have important consequences for specialized properties. The unique microenvironment of the brain, comprising myriad glial cells and neurons, creates specialized demands on the vasculature. Therefore, it stands to reason that cerebrovascular cell populations exhibit distinct and identifiable transcriptional programs to support these unique cell populations. Cerebrovascular cells within the vicinity of excitatory glutamatergic pyramidal neurons in the cortex are likely to have different properties compared to those in the striatum, which contain mostly inhibitory GABAergic medium spiny neurons. This, in turn, could lead to different functional consequences on hemodynamic responses due to the differences in neurochemical ligand and receptor combinations [230]. The concept of regional heterogeneity in cerebrovasculature molecular profiles is reinforced by the non-uniform yet stereotypic vascularization of the brain [35]. White matter regions are considerably less vascularized compared to gray matter regions, and cortical regions on average have greater density of blood vessels compared to some subcortical regions such as the striatum and hippocampus. Regional heterogeneity in the cerebrovasculature is also consistent with known gene expression differences within regions of the meninges and the choroid plexus [33, 194]. Most recently, work investigating molecular profiles within the median eminence, one of the circumventricular organs (CVOs), demonstrated not only differences in cerebrovascular gene expression profiles, but also morphological features in perivascular cell types [231]. CVOs, however, are known to lack extensive barrier properties and their blood vessel morphologies are distinct from BBB capillaries. Nevertheless, CVOs provide essential neurosensory and secretory roles, emphasizing the need to study cell type composition within these regions. Whether all CVOs exhibit the same cell type composition and molecular profiles or each exhibits distinct patterns, is currently not known but will likely be further investigated in the near future.

As an extension to regional heterogeneity, precise neurovascular interactions, particularly in non-cortical regions, is also poorly understood and will require future investigations to be well understood. Single cell genomic approaches allow for profiling of large numbers of cells but only at a single time point per sample. Temporal dynamics are not thus readily captured, but recent advances in two photon microscopy are yielding important insights into the functional properties of cerebrovascular cells and their interactions with the brain parenchyma [40, 46]. Furthermore, the capability of performing longitudinal studies allows for monitoring of functional changes with age and disease progression [232]. Unfortunately, two photon microscopy is limited by how deep light can penetrate into the brain, thereby precluding the study of subcortical structures such as the striatum, hippocampus, and thalamus. Alternative imaging modalities such as functional magnetic resonance imaging (fMRI) and positron emission tomography (PET) scanning allow for whole brain monitoring but lack the spatial resolution to interrogate changes at the cellular level. These technologies have provided important insights into functional changes that emerge throughout the cerebrovasculature in neurodegeneration and other diseases. Therefore, advances in live imaging technologies will greatly improve our capability to understand dynamic structures across brain regions at the cellular level.

## Conclusions

The clear evidence of cerebrovascular dysfunction across early stages of many neurodegenerative diseases, in addition to the expression of many disease relevant genes within cerebrovascular cell populations, strongly suggest that the cerebrovasculature plays an important role in disease pathogenesis and progression. From the neurodegeneration that is observed in rare genetic disorders linked to genes primarily expressed in the cerebrovasculature, it is clear that vascular dysfunction alone (absent primary causes in neurons) is sufficient to cause extensive and fatal neurodegeneration, but the extent to which cerebrovascular deficits contribute causally to common neurodegenerative disease mechanisms (in AD, PD, HD, and ALS; Fig. 2) remains to be resolved. Even in monogenic diseases such as HD, the causative gene’s (e.g. *HTT)* expression is detected within cerebrovascular cell populations, implying direct effects within these cell types. In combination with neuronal mechanisms, cerebrovascular deficits very likely enhance neurodegeneration and exacerbate phenotypes observed in these diseases.

Most current therapeutic approaches are focused on crossing the BBB to enter the brain and target delivery to neurons. To date, no attempt to directly target the cerebrovasculature as a disease-modifying intervention in neurodegeneration has been attempted. However, establishing how efficacious restoration of the cerebrovasculature alone or combination with neuronal-targeted therapies can be, will be important for the optimization of future therapeutic development. As the cerebrovasculature is more readily accessible than the brain parenchyma, it can be more easily targeted through systemic administration of gene therapy approaches. More importantly, the common findings of cerebrovascular dysfunction across all the major neurodegenerative diseases implies broad applicability of future cerebrovascular-targeted approach for multiple indications.

## Data Availability

Not applicable.

## Abbreviations

AAV: adeno-associated virus
AD: Alzheimer’s disease
ALS: Amyotrophic lateral sclerosis
ASO: antisense oligonucleotide
BBB: blood-brain barrier
BCSFB: blood-cerebrospinal cord barrier
CAA: cerebral amyloid angiopathy
CADASIL: cerebral autosomal dominant arteriopathy with subcortical infarcts and leukoencephalopathy
CARASIL: cerebral autosomal recessive arteriopathy with subcortical infarcts and leukoencephalopathy
CBF: cerebral blood flow
CNS: central nervous system
CSF: cerebrospinal fluid
CVO: circumventricular organ
DA: dopamine (dopaminergic)
DCE: MRI-dynamic contrast enhanced magnetic resonance imaging
ECM: extracellular matrix
FDA: Food and Drug Administration
fMRI: functional magnetic resonance imaging
FTLD: Frontotemporal lobar degeneration
GWAS: genome-wide association studies
HD: Huntington’s disease
ISF: interstitial fluid
MELAS: Mitochondrial encephalopathy, lactic acidosis, and stroke-like episodes
MS: Multiple sclerosis
MSN: medium spiny neuron
NGS: Next Generation Sequencing
NVC: neurovascular coupling
PD: Parkinson’s disease
PET: positron emission tomography
PFBC: Primary familial brain calcification
PVF: perivascular fibroblast
PVM: perivascular macrophage
SCA: Spinocerebellar ataxia
sc/snATAC: seq-single cell/nucleus assay for transposase-accessible chromatin sequencing
sc/snRNA: seq-single cell/nucleus RNA sequencing
SMC: smooth muscle cell
SNP: single-nucleotide polymorphism
SNpc: substantia nigra pars compacta
TfR: transferrin receptor
TRAP: Translating Ribosome Affinity Purification
VEN: von Economo neuron
VCI(D): vascular cognitive impairment (and dementia)
